# International medical electives in Sub-Saharan Africa: experiences from a 19-year NGO-driven initiative

**DOI:** 10.1186/s12909-023-04154-y

**Published:** 2023-03-27

**Authors:** Gianluca Quaglio, John Bosco Nsubuga, Donald Maziku, Ademe Tsegaye, Nicoletta Parise, Chiara Cavagna, Peter Lochoro, Maria Grazia Strepparava, Liviana Da Dalt, Sam Okori, Alessandra Gatta, Adrien Mbiya Kamunga, Giovanni Putoto

**Affiliations:** 1 Medical Preparedness and Crisis Management Unit (MPCMU), Directorate-General for Personnel, European Parliament, Rue Wiertz, 60, B-1047 Brussels, Belgium; 2 Operational Research Unit, Doctors with Africa Cuamm, Padova, Italy; 3 Saint Kizito Hospital, Matany, Uganda; 4 Tosamaganga Hospital, Iringa, United Republic of Tanzania; 5 Saint Luke Hospital, Wolisso, Ethiopia; 6 Department of Statistical Sciences, Padova University, Padova, Italy; 7 Doctors with Africa Cuamm, Kampala, Uganda; 8 Clinical Psychology Unit, San Gerardo Hospital, Monza, Italy; 9 School of Medicine and Surgery, University of Milano-Bicocca, Milan, Italy; 10 Division of Paediatric Emergency Medicine, Department of Women’s and Children’s Health, University of Padova, Padova, Italy; 11 Aber Hospital, Aber, Uganda; 12 Doctors with Africa Cuamm, Pujehun Hospital, Pujehun, Sierra Leone

**Keywords:** Medical elective, International health, Sub Saharan-Africa, Global health

## Abstract

**Background:**

Mainstream medical education remains largely focused on national health issues. Therefore, in order to expose medical students to international health issues, it is beneficial to facilitate international medical electives.

**Methods:**

This article describes the Junior Project Officer (JPO) program, a medical experience based on clinical electives in Sub-Saharan Africa, supported by a Non-Governmental Organisation (NGO). Residents spend 6 months as part of a multidisciplinary medical team in Africa. A post-elective online survey was administered to all who participated in the program in the period 2002–2020. The questionnaire comprised three domains: (i) general and pre-departure information; (ii) the experience; (iii) the post-experience.

**Results:**

Questionnaires were received from 157/241 subjects, a response rate of 65%. The most common specialties were pediatrics, public health, and internal medicine. Of all, 87% carried out clinical activities; 45% also worked in the management of health services, and 60% carried out research activities. About 64% reported difficulties linked to a lack of equipment, different ways of working (57%), and exposure to situations for which they did not feel technically prepared (56%). In 25% of cases, residents reported that their school’s attitude to their doing the elective was not positive: upon their return, over 50% felt that their experience was not sufficiently valued by their institution. Respondents considered the experience important for professional and personal growth (93% and 80% respectively ). Forty-two participants (27%) reported that the experience had a significant impact on their future career choices.

**Conclusion:**

Despite the difficulties encountered, a well-structured experience in international health can have a positive impact on residents, professionally and personally. Key factors behind the positive outcomes are the substantial length (6 months) of the experience, and the long term working relationships between the sending and receiving institutions. The schools in Italy that provide the students for the electives need to see more evidence that international electives are worth the investment.

**Supplementary Information:**

The online version contains supplementary material available at 10.1186/s12909-023-04154-y.

## Background

The provision of global health training through international clinical experiences for medical students is now a necessity. This may be traced back to two fundamental causes: the first is that globalization affects many aspects of public health, as has been underscored by the COVID-19 pandemic [1]. The second is that mainstream medical education is substantially focused on national and not global health issues [2, 3]. An increasing number of medical students are undertaking electives in low- or middle-income countries (LMICs) [4]. Medical experiences in LMICs give hands-on understanding of working in under-resourced conditions and in a different cultural environment. Medical electives increase diagnostic skills in conditions where medical technology is often non-existent. They bring a greater appreciation for public health interventions on community health [5–7]. However, medical electives have brought to light multiple concerns, including navigating an unfamiliar health system with different workflows [8, 9], health risks associated with electives [8, 10], and possible doubts about how meaningful and positive the impact on host communities may be [11–14]. International guidelines have been developed for best practices in electives. These guidelines aim to better understand local cultures, ethical implications, personal motivations, and to ensure that elective activities improve local health capabilities [15–17].

Global health is a concept and reality that has emerged almost entirely from western institutions. This base in the traditions of the west has even led to the claim that ‘global health’ is neo-colonialist [18]. This has led to current international electives facing rigorous monitoring and criticism [19–21]. Cole noted how international medical electives are carried out predominantly by privileged groups, which assume that, ‘they can make a difference without understanding the complexities of context of those being helped’ [22], perpetuating the notion that, ‘some care is better than none’ [23]. Sullivan observed that electives, ‘commonly arrive lacking contextual and cultural knowledge, let alone specific notions of what precisely they will do. Volunteers thus arrive and interact in an ad hoc fashion’ [21]. Dowell, and Merrylees (2009) [24] pointed out that, ‘electives in developing countries tend to be popular and memorable, but are generally so unstructured that they raise a number of moral issues and perhaps fail to fulfil all the educational opportunities they seek to offer’. These criticisms highlight the need to ensure that international electives should be well prepared for the experience. In addition, critics require that elective activities meaningfully mitigate against the global and local factors that drive imbalances in terms of knowledge, resources, and level of healthcare. Instead of focussing only on biomedical and clinical capacity, global health practices should adopt multidisciplinary approaches, acknowledging social and economic inequity at community and global level [25].

In order to develop a well-structured elective experience, Dowell and Merrylees observed it is important to establish, ‘institutional partnerships between the sending and receiving institutions’. They also emphasise that, ‘the continuity of student presence provided by a partnership allows more comprehensive student preparation’, and that the preparation of students for electives, ‘is easier to organise and of more direct relevance for groups of students who are going to the same place as part of an institutional partnership’ [24]. In short, a stable relationship with the host institution helps prepare residents for the elective experience and can lessen the potential concerns relating to host institutions in LMICs [26, 27]. We reviewed over two-hundred articles on international medical electives, and to the best of our knowledge, there has been no systematic analysis of how institutions that provide residents begin their involvement with international medical electives. Typically, providers engage through personal connections and by joining faith-based medical mission teams. Academic institutions more often begin their involvement by establishing or participating in bilateral academic partnerships [9].

This paper sets out the experience called the Junior Project Officer (JPO), driven by the Non-Governmental Organization (NGO), Doctors with Africa - CUAMM (DwA) [28]. A resident international elective requires that the time needed for participants to adapt to the hosting site be carefully balanced with the issues arising from time spent away from the home institution. The JPO offers doctors-in-training the chance to undertake a six-month health elective in Sub-Saharan Africa. The six-month elective allows residents enough time to adjust psychologically to a challenging environment, and to better understand the different aspects of global health related to the experience. In its 19 years of activity, the JPO has collaborated with 37 Italian medical schools. With 12 of these, the relationship is governed by a memorandum of understanding. A memorandum of understanding has also long been established by DwA with each of the African health facilities hosting JPO residents. The experience is clinically focused, and includes public health practices and research components. Continuity of care between hospitals, peripheral health centres, and communities is key to the organization’s activities. Accordingly, in addition to hospital work, residents carry out visits to peripheral health centres and communities. These activities are mediated by government health staff (District Health Management Teams and Community Health Workers). The meetings with communities, especially with women, are aimed at learning about healthcare-seeking behaviours and the cultural, geographical, and financial barriers that prevent populations from accessing health services. DwA also organises pre-graduate medical elective international experiences, as described elsewhere [29].

DwA is the first NGO working in the international health field to be recognised in Italy and is the largest Italian organisation for health promotion in Africa. DwA is present with offices and staff in eight Sub-Saharan African countries; Angola, Central African Republic, Ethiopia, Mozambique, South Sudan, Uganda, Sierra Leone, and Tanzania [28, 30]. At clinical level, the organisation supports 23 main hospitals and 761 health facilities. The major areas of intervention are maternal and child health, nutrition, infectious diseases, and chronic diseases. DwA provides training support to one university and four schools of nurses and midwives. In 2021, DwA had 4,518 health professionals and technicians on the ground. The organisation works with a long-term development perspective, often undertaking projects that will last for decades. It receives national and international private and public funding.

The 19 years’ experience of the JPO is reported in this paper, using the results of a survey distributed to all residents who participated in the JPO.

### Managerial and recruitment process for JPO residents

The screening process for the selection of residents for the JPO consists of several steps aiming at assessing motivation, cultural sensitivity, and commitment to working in a setting with limited resources. Prospective JPO candidates fill in an application form, eliciting basic information, including professional profile, academic record, and reasons for participating in the elective. Residents are required to attend pre-departure training courses organised by DwA, during which the selection of candidates is finalised. Over the 19 years of the project, the training has been progressively revised, based on the feedback from the participants and from the African hospitals hosting the residents. At present, it consists of four main sections. The first is devoted to the objectives of the JPO. The second section provides information management on specific clinical fields, along with concepts of hospital management and public health in poor resource settings. The third section aims at educating the participants regarding the socio-cultural context of the host country. This preparation is tailored to each location, and is generally taught by people from the relevant countries. It includes information on how historical factors, such as colonisation and exploitation of resources, have affected health services. In addition, the third section contains a module dedicated to health. This follows the LEARN medical anthropology framework (Listen, Explain, Acknowledge, Recommend, Negotiate) [15]. The fourth section provides bureaucratic and practical information. The trainers are health professionals with previous experience in LMICs.

Before departure, the most suitable location in Africa is identified and a personalised job description is prepared for each resident, in line with the needs of the host institutions and the training objectives of the specialty school. All the locations where DwA works are potential destinations, provided that the presence of the tutor as well as logistical and safety conditions are guaranteed. These conditions may vary over time depending on the evolution of DwA’s intervention and the situations in the countries where it operates.

Upon their return, residents are asked to complete an evaluation form and attend debriefing sessions with DwA staff. Debriefing provides an opportunity for residents to reflect on the frequently mixed emotions experienced, and on the challenges faced, thus allowing for further personal and professional development. Often, residents are invited to share their experience during an ad hoc seminar in their school of speciality and encouraged to elaborate upon their experience through reflective writing, which is published on the DwA website.

## Methods

### Study design

The study design was a cross-sectional online survey.

### Setting and on-site activities

There are 8 countries and different potential destinations: Angola (Chiulo hospital, Luanda); Ethiopia (Wolisso hospital); Mozambique (Beira central hospital); Central African Republic (Bangui hospial); Sierra Leone (Freetown hospital and hospital and health centres in the Pujehun district); South Sudan (Yirol hospital and Lui hospital); Tanzania (Tosamaganga hospital); Uganda (Aber hospital and Matany hospital). Travel costs are incurred directly by residents (Table 1). DwA provides insurance coverage, accommodation and logistical support.

**Table 1:** Major sites of activities of the JPO programme

Major sites of electives	Beds	Out-patients visits	In-patients admissions	Health location
Ethiopia: Saint Luke H^1^, Wolisso	200	80.282	12.183	Regional referral H, urban area; Community; Districts
Central African Republic: Bangui Pediatric H	257	71.065	16.309	National referral H, urban area
Mozambique: Beira Central H	823	126.150	17.159	National referral H, urban area
South Sudan: Yirol H and Lui H	105	54.470	10.391	National referral Hs, urban area; Community; Districts
Angola: Chiulo H, Luanda	234	25.055	4.510	District referral H, rural area; Community; Districts
Uganda^2^: Aber H and Matany H	428	64.948	26.259	District referral Hs; rural area; Community; Districts
Tanzania: Tosamaganga H	165	31.963	6.354	District referral H, rural area; Community; Districts
Sierra Leone^2^: maternity H, Freetown and H in the in Pujehun district	184	20.162	12.501	National and district referral hospitals; urban and rural areas; Community; Districts

^1^ H: Hospital; 2: cumulative data

In Africa, the residents are required to participate in all the daily activities of the service, including patient visits, case discussion, making diagnosis and management of illnesses, meetings with nurses and families, and on-site formularies. This brings familiarisation with local disease patterns and the limitations of local health resources. The clinical care process is mediated by the local health staff in charge, such as medical doctors, nurses, and hospital managers. The main way of communicating with the patients and their relatives is through the national language (e.g. Kiswahili, English, French, Portuguese, etc.). At the community level, where people commonly speak local dialects, language barriers are overcome by local district health managers and community health workers. Senior doctors act as tutors, helping residents to deal with the difficulties that are inevitably encountered in this type of situation. Tutors are involved in drafting the training objectives approved by the University, providing monthly feedback and a final evaluation of each resident’s activity. Since the start of the project, over 50 professionals have been tutors, both expatriates and locals, most of them being head of a hospital ward or department, and specialised in the same or a closely related speciality as the resident. Typically, they have more than 10 years of experience as consultants. Motivation for the tutors is multifaceted. There are no financial incentives, however teaching trainees brings its own rewards. In addition, tutors familiarise themselves with new procedures, and can network with international academia. Also of value is that residents help share the burden of clinical work.

### Sample, eligibility and data collection

The survey, carried out in July 2021, was electronically distributed to all subjects who participated in the JPO program during the period 2002–2020. Participants were informed that the data would be used exclusively for research purposes and that the data were collected in conditions of complete anonymity. The questionnaire encompassed three sections: (i) general and pre-departure information; (ii) the experience; (iii) the post-experience (Annex I). The study was approved by the Ethics Committee of DwA.

### Statistical analysis

All statistical analysis was performed using SPSS v.26. The variables were of two types: categorical variables, such as characteristics of individuals or of their experience (year of departures, destination, main motivation, etc.); ordinal variables (values from 1 to 10), used by participants to assess their professional or training experience. Categorical variables were recapitulated using frequency tables (percentage distribution). Ordinal variables were summarised using quartiles. Bivariate analyses were carried out to identify associations between variables (or differences) between groups of respondents. In order to evaluate the association between categorical variables, cross tables and Pearson’s chi-square test were conducted. For example, analysis was conducted to see if there were differences in different periods (2002–2013 vs. 2014–2019) or in different working areas (medical vs. surgical). A number of paired t-tests was used to test if the means of two paired measurements (pre-departure score and post-departure score) were different. A value of p < 0.05 was considered significant.

## Results

### Progressive increase in the number of participants

Over the period of the study, the JPO program received an average of 30 requests per year, and there were on average 12 departures per year. However, the number of residents doing a medical elective progressively increased over the years. In the first 5 years of the project, there was an average of 7 applications per year: this increased to 56 per year in the last 5 years of the project. During the course of the project, there was a corresponding increase in the number of departures, from an average of 4 per year in the first five years to 27 per year in the final five years (data not shown).

### General and pre-departure information

Of the 241 subjects who took a medical elective during the study period, 157 completed the survey (65% response rate). The majority of the questionnaires (54%) were completed by those who had had the elective experience in the last 5 years of the study period. The majority of participants were female and carried out the electives at Wolisso, Tosamaganga, and Beira hospitals. The most frequent specialties were pediatrics, public health, internal medicine and subspecialties. At the time of departure, most participants were in year 4 or year 5 of their residency (Table 2).

**Table 2:** General and pre-departure information

Topic				No	%
**Gender**					
Female				116	74%
Male				41	26%
**Year of departure**					
2002–2007				20	13%
2008–2014				41	26%
2015–2020				96	61%
**Post-graduate year of residency**					
PGY-1				3	2%
PGY-2				5	3%
PGY-3				36	23%
PGY-4				71	45%
PGY-5				42	27%
**Specialisation**					
Pediatrics				47	30%
Hygiene and preventive medicine				25	16%
Internal medicine and subspecialties				25	16%
General surgery				19	12%
Gynecology and obstetric				17	11%
Infectious diseases				14	9%
Emergency medicine				7	4%
Anasthesia				3	2%
**Location of the elective**					
Wolisso (Ethiopia)				28	18%
Tosamaganga (Tanzania)				27	17%
Beira (Mozambique)				27	17%
Chiulo (Angola)				19	12%
Matany (Uganda)				12	8%
Aber (Uganda)				9	6%
Freetown (Sierra Leone)				8	5%
Others				27	17%
**Motivations**					
To serve less privileged populations				91	58%
To enhance my professional experience				47	30%
Others				19	12%
**Effectiveness of pre-departure training**					
Yes				118	75%
No				39	25%
**Response of the school to the JPO**					
Very well				52	33%
Moderate				66	42%
Neutrally				27	17%
Not well				12	8%
**In Africa before**					
Never				68	43%
As a volunteer				61	39%
As a tourist				28	18%
Activity carried out in current work*					
Clinical activity				126	80%
Research activity				33	21%
Organisation of services and health planning				25	16%
Didactic and training activities				25	16%
**Current place of work**					
Italy				141	90%
Abroad				16	10%

* More than one answer was possible

The desire to serve less privileged populations and to improve one’s professional preparation were the major reasons behind the decision to undertake the experience. There were no statistically significant variations between the motivations and the year of the experience.

As a whole, the support received in preparing for departure is evaluated positively by 75% of respondents. Further analysis of the pre-departure training shows that the major criticisms were related to the preparation for clinical activities, with a lack of specific information related to the location (hospital) of medical elective; the available resources for clinical care and the internal organisational arrangements (data not shown).

About one in 4 respondents (25%) stated that their medical school of specialisation did not welcome the choice to participate in the JPO.

### The experience

Most of the activities were carried out in hospitals or community health centres (87% and 31% respectively). Clinical activities were mainly done in hospital, with in-patient and out-patient levels at 76% and 43% respectively. The educational and training activities were carried out mainly with the general population (93%) and with community health workers (87%), while the organisation of health services was carried out in health centres and villages (31% and 15% respectively) (Table 3).

**Table 3:** The experience

Topic	No	%
Health location of the elective*		
Hospital	125	87%
Community	49	31%
District	22	14%
Others	9	5%
Clinical activities*		
No	20	13%
Hospital	119	76%
Outpatient clinic	68	43%
Emergency	41	26%
Others clinical activities	30	19%
Didactic/training activities*		
No	30	19%
General population	146	93%
Community health workers	137	87%
Doctors and/or clinical officers	99	63%
Nurses	55	35%
Other didactic/training activities	143	91%
Organisation of health services*		
No	86	55%
Health centres	49	31%
Villages	24	15%
Districts	22	14%
Other organisational services	16	10%
Research activities*		
No	63	40%
Research for the specialisation thesis	52	33%
Research for posters/abstracts/presentations	24	15%
Research for articles in peer review journals	22	14%
Other research activities	27	17%

*More than one answer was possible

**Fig. 1:**
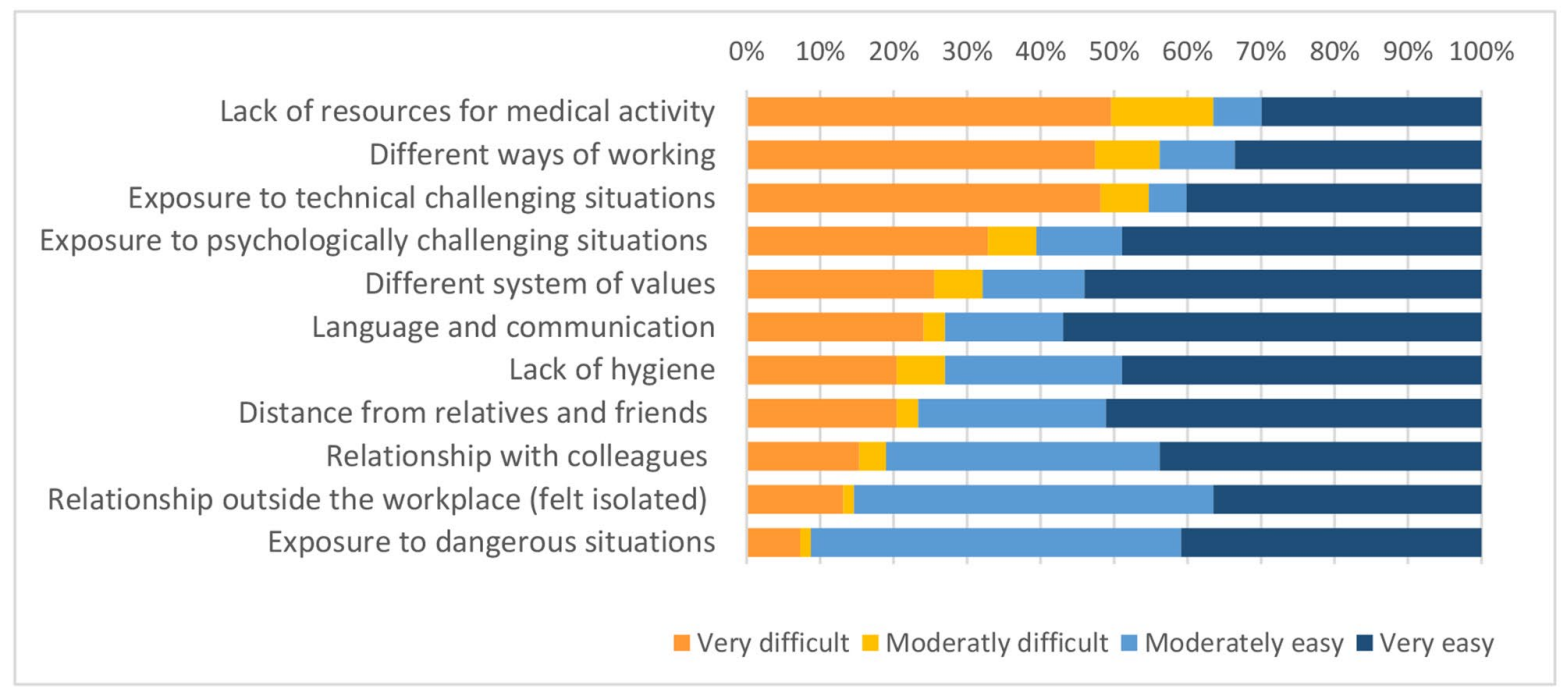
Obstacles reported during the medical elective

About 64% reported difficulties linked to a lack of equipment (materials, devices, drugs, etc.), different ways of working (57%), and exposure to situations for which they did not feel technically prepared (56%). The share of those who reported difficulties due to exposure to situations for which they were not psychologically prepared was also significant (39%). A minority had difficulties in integrating with colleagues (19%) and in relationships outside the workplace (14%) (Fig. 1). A further analysis was made to see if there was a relationship between any of these obstacles, the host institutions, the host countries, and the working area (divided between medical and surgical). No statistically significant differences emerged with the host institutions and host countries. The obstacle ‘exposure to technically challenging situations’ was reported by 85% of those who worked in the surgical area and by 49% of those who worked in the medical area (p < 0.01). For the other obstacles, no significant differences between the two groups were identified.

### The post-experience

Training objectives included management of the clinical field in poor resource settings, understanding health inequities and other public health issues, improving health services management, and achieving professional and personal growth. In total, 58% of respondents felt that the training objectives were fully achieved. Only 2% stated that they were not. A significant percentage (40%) declared that the training objectives were only partially achieved. Notably, trainees considered the JPO experience important for professional and personal growth (respectively 93% and 80% of respondents). The analysis of these responses in relation to the period of departure and the working area (medical versus surgical), revealed no statistically significant differences.

Forty-two participants (27%) reported that the experience had an impact on their future career choices.

The experience positively influenced the medical approach of the residents. Respondents say they gained in terms of autonomy at work and self-confidence (79%) and in resilience (77%) (Table 4). The analysis of these responses in relation to the period of departure and the working area (medical versus surgical), revealed no statistically significant differences.

**Table 4:** The post-experience

Topic	No	(%)
**Achievement of formative objectives**		
Fully	91	58%
Partially	63	40%
No	3	2%
**Contribution of elective on personal growth**		
Very much	146	93%
Fair	9	6%
Slight	2	1%
None at all	0	0%
**Contribution of elective on professional growth**		
Very much	125	80%
Fair	25	16%
Slight	5	3%
Not at all	2	1%
**Impact on future career choices**		
Yes	42	27%
No	115	73%
Impact of elective on medical practices*		
Autonomy at work and self-confidence	124	79%
Resilience	121	77%
Empathy with the patient	104	66%
Patience	89	57%
Aptitude to work with others	85	54%
Respect for others	78	50%
**Valued by your school of specialisation upon returning**		
Very much	20	13%
Fair	46	29%
Slight	55	35%
Not at all	36	23%
**Maintained contact with the hospital in Africa**		
Yes	78	50%
No	79	50%
**Other experiences in LMICs after the elective**		
Yes	46	29%
No	111	71%
**Other work experiences in LMICs in the future**		
Very much	52	33%
Fairly	89	57%
Slightly	14	9%
Not at all	2	1%
**Maintained contact with DwA after the elective**		
Yes	143	91%
No	14	9%

*More than one answer was possible

**Fig. 2:**
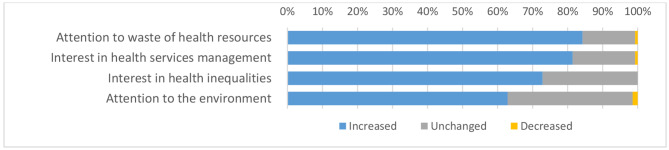
Residents’ interest in health inequalities, commitment to the environment, awareness of wasting health resources, and interest in health services management (post-experience data)

Other questions explored how the experience affected residents’ interest in health inequalities, commitment to reducing environmental damage, awareness of wasting health resources, and interest in health services management. These questions compared pre and post-experience data (Fig. 2): the comparisons were statistically significant (p < 0.01).

Subsequent to their elective, 29% have spent additional time in a medical setting in Africa, and 33% consider having other similar experiences in LMICs in the future. After their elective, 50% of residents have maintained contact with the African setting; 91% have maintained some form of contact with DwA (training, activities of cooperation, etc.).

## Discussion

### Principal findings

Mutchnick et al. [2] observe, ‘living in alternative social environments creates an educational experience unmatched in any textbook or classroom exercise’. Residents in this study reported benefits from their experience which fall into three categories. First, at the professional level, residents declared that they returned home with an improved medical self-confidence, a greater sense of empathy towards patients, and an increased awareness of problems concerning the use of resources. This contributed to better cost-effectiveness and resource utilisation on return home [27, 31, 32]. Second, at the educational level, residents reported a deeper interest in health services management, and a greater appreciation of issues such as health inequalities, environmental damage, and the wasting of health resources. Third, the electives resulted in long-term behavioural change among residents. Consistent with prior reports, the experience seemed to influence the careers of a significant percentage of residents, increasing their interest in public service [7, 27, 32].

### A stable relationship between sending and hosting institutions

As observed by Ackerman [33], ‘on-site supervisors, the back-bone of most electives, are only possible with a reciprocal, long-term relationship either through a local university and medical school, a Nongovernmental Organization (NGO), or an International Nongovernmental Organization (INGO) working in the area. The educators must ensure that the host organization is appropriately integrated into the community and that community goals are at the forefront’. Willott et al. [34] highlight that, ‘students frequently want to be able to decide for themselves where they go and how they spend their electives, but this may not be what is best for hosting institutions, nor for global health more generally’. As pointed out by Edwards et al. [35], a major concern regarding medical electives is that students may practice, ‘beyond their competence, to their own and their patients’ detriment. This may be more common in developing countries where supervision is scant and students may assume that limited health care resources justify their adopting roles or performing procedures which would be restricted to fully trained staff at home’. These reflections highlight the benefits of an experience like the JPO, which is organised and implemented by an NGO with long-term working relationships with the African populations and is well integrated into the community. The project is carried out in health facilities where DwA staff have been working for many years. Having well-known locations for electives reduces the potential risks connected with this type of experience, and better ensures a satisfactory level of supervision, a lack of which is a serious problem in many similar experiences [36]. A stable relationship between sending and hosting institutions may take several years to establish. The positive results of the JPO program were made possible by the continuous on-site presence of DwA. As observed by Luchett et al., ‘this continuity provides a larger framework for residents so that they can contextualize their knowledge and understand their role’ [37].

### Length of experience and obstacles encountered

A resident international elective requires a balance between the time required to adapt to the hosting site and the organisational constraints associated with being away from the resident’s home institution. Drain et al., suggest that residents in international rotations should spend at least six weeks working at the host institution [5]. It is a unique feature of the JPO project that the residents spend 6 full months on the elective. This gives JPO residents a much better sense of global health than is possible from most international rotation programs, which typically last between one and three months. Furthermore, this time frame is more likely to accommodate a more thorough psychological adjustment to a new and challenging environment. The six-month period is counted as equal to any other mandatory rotation that residents undertake during their training.

Consistent with prior reports, the major obstacles and frustrations reported are related to the working conditions: limited resources (e.g. fewer human resources, lack of medication and equipment, a lack of laboratory and imaging support, etc.), different ways of working (e.g. team structures or clinical roles that differ from what residents are used to), and clinical situations for which residents were technically unprepared (e.g. managing unfamiliar medical conditions) [9, 38]. The exposure to situations for which residents were not psychologically prepared was also significant. The emergence of mental health problems arising from coping with culture shock and working in problematic settings, often with high morbidity and mortality, are recognised elsewhere [9, 38, 39].

A less direct problem is how the providing school of specialisation reacted to the resident’s departure and return. In 25% of cases, residents reported the school’s response to departure was not positive. In addition, over 50% reported that upon their return their experience seemed insufficiently appreciated by their institution. These data must not be overlooked. This may be explained by the shortage of health personnel in Italy and many European Member States, which is an ongoing barrier to pursuing international clinical rotations. The providing schools of specialisation need to see more evidence that it is worth their investing in international electives. A more rigorous evaluation of the effectiveness of elective experience is needed to demonstrate the value added to medical training [40], and what impact it may have on the communities and institutions involved [41]. Long-term follow-ups of elective participants after medical school in relation to their career choices (e.g., type of medical practice, career developed in public or private sectors) can also persuade medical schools that it is worth their investing in medical electives [32].

### Activities in research and public health

Conducting research in Africa is not easy, for several reasons. Health institutions in Africa are often reluctant to support research efforts. This hesitation may stem from difficulties in data gathering, chronic shortcomings in clinical service provision, and fear that the research results may expose poor performance [42]. Additional frustrations arise from difficulties in understanding whom the research activities most benefit - the host institution in Africa, or the academic home institution. Over recent years, research has gained increasing importance in the DwA organisation and the JPO program. Research activities are embedded in the interventions meant to improve access and quality of care, or in stand-alone projects, which are promoted by DwA and the host institutions. About a third of the residents develop a research topic in line with their own specialty thesis, using locally available data. All JPO research-based initiatives must obtain both home and host site approval, as well as ethical approval from the ethics committees of the host institution and national research authorities. In addition, presentations and articles must recognise the contributions from African partners. In the years 2018–2020, DwA published 91 articles in peer-reviewed journals: of these, 44 had medical electives as co-authors [43]. While research collaboration between residents and host institutions is promising, the process is still far from optimal.

Residency programs often have difficulty teaching issues such as cost awareness and health inequity. To address the issue of paucity of resources, problem-solving ability was developed by the residents through alternative diagnostic and therapeutic solutions. Local resources including semeiotic knowledge, simple point of care testing, basic drugs regimen, and essential care for critical patients, were the main factors affecting residents’ autonomy, self-confidence, and resilience. Since the clinical services are not provided for free, the residents developed a “cost awareness” attitude, searching out the most beneficial and cost/effective clinical pathways. This also contributed to seeing the severity of inappropriate care in western countires, where the wasting of health resources is significant and even dangerous. For instance, over prescription of antibiotics has led to increased antimicrobial resistance [44]. Patient delay causes severe, often fatal medical complications. To respond to this common phenomenon in Africa, residents developed a capacity to act quickly in dealing with critical patients, and increased their resilience in working under stressful conditions. Through attending death audit sessions, they see that to assure continuity of care, it is essential that health systems operating at different levels are sufficiently interconnected.

The interconnection of climate change and health needs to be addressed at global and local levels. International electives make residents confront environmental issues with an immediacy not possible in the home environment. For example, in 2019, when Mozambique was hit by Cyclone Idai, residents remained in the field, and provided humanitarian and clinical support to Beira Hospital and surrounding HIV health services [45, 46]. When Angola was hit drought, exacerbating child malnutrition, JPO residents similarly remained in post [47]. Such experiences make residents more aware of the interconnectedness of global environmental and health issues.

### Study limitations

Although common to other reports of this type, it cannot be overlooked that this study has several shortcomings. Although the majority of questionnaires were completed by those who underwent the JPO experience in recent years (2016–2020), there was significant variation in the time elapsed between the experience in Africa and the administration of the questionnaire. The JPO is carried out in eight countries with over a dozen potential destinations. Significant disparities exist on economic activities, welfare, health policies and social indicators across these countries. Furthermore, geographic settings are dynamic, constantly evolving in terms of socio-economic and political aspects, access to basic services (such as education and infrastructure) and public health policies. Therefore, it is challenging to examine whether and how changes in the African settings might have influenced the experiences of the residents during the nearly two decades of the JPO program. While this was not the goal of this study, it is an intrinsic limitation of it. A further limitation of this study is its retrospective design, with no comparison control group. The study utilised a self-administered questionnaire, without measures to address potential self-reporting bias. Although hosting institutions generally gave positive feedback, the information has not yet been systematically collected. An additional limitation is related to the lack of systematic collection of the feedback provided by residents after their experience. There is a need to better quantify the impacts of JPO on clinical knowledge and patient care. Future objectives of the JPO are to collect structured feedback from local communities, including additional outcome measures, other than self-reports, focusing on provider behaviour, clinical knowledge, and patient care.

## Conclusion

This study reveals the results of structured and ongoing medical elective experiences carried out by an NGO in Sub-Saharan Africa. The ever-increasing globalisation deepens the need for physicians to be culturally broadminded and to be effective communicators. International electives are an important means towards engaging with the diversity of patients physicians will encounter along their careers [27, 48]. The JPO has enabled elective experiences to people from 37 Italian universities (there are 43 faculties of medicine in Italy). The program seems to be making up for the lack of international experience in sub-Saharan Africa offered by Italian universities [49]. This is confirmed by the steadily increasing number of agreements between DwA and Italian universities. Further, this work highlights the need for Italian academic institutions and medical schools to nurture a medical education approach that is able to train doctors internationally competent and with a professional profile sensitive to global health issues. Ensuring adequate supervision and stable partnerships with hosting sites in Africa is mandatory, in order to respond to the unique logistic and ethical challenges that arise. The experience may present residents with practical and emotional challenges. A stable relationship with host institutions can also provide a continuous chain of residents over many years, contributing to the capacity building of the host institutions. Our findings support previous research about the value of international electives for residents. The mission of the medical school should be not only to train good clinicians, but also to be more community oriented, reconciling the health needs of the individual and the community.

## Electronic supplementary material

Below is the link to the electronic supplementary material.

Questionnaire of the study

## Data Availability

The datasets used and/or analysed during the current study are available from the corresponding author on reasonable request. The questionnaire used in the study was developed specifically for this study (in Italian). An English version is available as a supplementary file.

## Abbreviations

LMICs: Low- or Middle-Income Countries
JPO: Junior Project Officer
NGO: Non-governmental Organization
DwA: Doctors with Africa
CUAMM: Collegio Universitario Aspiranti Medici Missionari
INGO: International Nongovernmental Organization
