# Supplementary material for: International medical electives in Sub-Saharan Africa: experiences from a 19-year NGO-driven initiative
Source: BMC Med Educ. 2023 Mar 27;23:184. doi: 10.1186/s12909-023-04154-y (PMC10041499; doi:10.1186/s12909-023-04154-y)
Supplement: Supplementary file 1 — Questionnaire of the study [file 12909_2023_4154_MOESM1_ESM.docx]

**International medical electives in Sub-Saharan Africa:**

**Lessons from a 19-year NGO-driven initiative**

Gianluca Quaglio, John Bosco Nsubuga, Donald Maziku, Ademe Tsegaye_,_ Nicoletta Parise, Chiara Cavagna, Peter Lochoro, Maria Grazia Strepparava, Liviana Da Dalt, Sam Okori, Alessandra Gatta, Adrien Mbiya Kamunga, Giovanni Putoto

**Questionnaire of the study**

| 1. **GENERAL AND PRE-DEPARTURE INFORMATION** |
| --- |

**1.1 Gender**

Male; Female

**1.2 Year of departure**

**1.3 Post-graduate year of residency at the time of departure**

1st; 2nd; 3rd; 4th; 5th

**1.4 What was your specialisation?**

**1.5 At the time of departure, in which University were you enrolled?**

**1.6 Was your decision to leave positively received by the management of your school of specialisation?**

Very well received; Moderately well received; Neutrally; Not well received

**1.7 Was the experience with the JPO project your first time in Africa?**

Yes; No, I have been there as a tourist; No, I have been there as a volunteer

**1.8 What was your main motivation for being part of this experience?**

*[Open question].*

**1.9 What are your main activities in the medical profession currently?**

Clinical activity; Research activity; Organisation of services and health planning; Didactic and training activities

**1.10 What is your current place of work?**

Italy; Abroad

**1.11 In general, was the pre-departure training satisfactory in general?**

Yes; No

**1.12 In more detail, was the pre-departure training satisfactory, in relation to the factors listed below?**

|  | Not at all | Slightly | Fairly | Very much |
| --- | --- | --- | --- | --- |
| Clinical work information |  |  |  |  |
| Socio-cultural context |  |  |  |  |
| Educational objectives |  |  |  |  |
| Bureaucratic procedures |  |  |  |  |

| 1. **THE EXPERIENCE** |
| --- |

**2.1 In which location did you have the experience in Africa?**

Angola (Chiulo hospital, Luanda); Ethiopia (Wolisso hospital); Mozambique (Beira central hospital); Central African Republic (Bangui hospial); Sierra Leone (Patient centred medical home, Freetown and hospital and health centres in the Pujehun district); South Sudan (Yirol hospital and Lui hospital); Tanzania (Health centres in Shinyanga region and Tosamaganga hospital); Uganda (Aber hospital, Angal hospital, Arua hospital and Matany hospital).

**2.2 Where did the JPO experience take place?**

Hospital; District; Community; Other

**2.3 Have you performed clinical activity? If so, where?**

No; Outpatient clinic; Hospital ward; Emergency; Other health structures

**2.4 Have you performed organisation of health services activity? If so, where?**

No; In health centres; In the villages; In the districts; Other health services

**2.5 Have you performed didactic/training activities? If so, to who?**

No; Doctors and/or clinical officers; Health workers; Community health workers; To the population; Other training activities

**2.6 Have you performed research activity? If so, which one?**

No; Research for the specialisation thesis; Research for posters/abstracts/presentations; Research for articles published in peer review journals; Other research activities

**2.7 What were the main obstacles you have encountered during the experience?**

|  | Very easy | Moderately easy | Moderately  difficult | Very difficult |
| --- | --- | --- | --- | --- |
| Distance from relatives and friends |  |  |  |  |
| Language and communication |  |  |  |  |
| Different system of values |  |  |  |  |
| Different ways of working |  |  |  |  |
| Lack of resources for medical activity |  |  |  |  |
| Lack of hygiene |  |  |  |  |
| Exposure to psychologically challenging situations |  |  |  |  |
| Exposure to technical challenging situations |  |  |  |  |
| Exposure to dangerous situations |  |  |  |  |
| Difficult relationship with colleagues |  |  |  |  |
| Difficult relationship outside the workplace (felt isolated) |  |  |  |  |

| 1. **POST-EXPERIENCE** |
| --- |

**3.1 Have the training objectives of the experience, set at the start, been achieved?**

Fully; Only partially; No

**3.2 Has the experience changed the way you approach the following aspects of the medical practice?**

|  | Decreased | Unchanged | Increased |
| --- | --- | --- | --- |
| Autonomy at work and self-confidence |  |  |  |
| Empathy with patients |  |  |  |
| Patience |  |  |  |
| Resilience |  |  |  |
| Respect for others |  |  |  |
| Aptitude to work with others |  |  |  |

**3.3 Has the experience had any impact on your future professional career choices?**

Yes

No

**3.4 How much has the experience contributed to your personal and professional growth?**

|  | Not at all | Slightly | Fairly | Very much |
| --- | --- | --- | --- | --- |
| Professional growth |  |  |  |  |
| Personal growth |  |  |  |  |

**3.5 Has the experience increased your interest in the following issues?**

|  | Not at all | Slightly | Fairly | Very much |
| --- | --- | --- | --- | --- |
| Health inequalities |  |  |  |  |
| Your commitment to the environment |  |  |  |  |
| Your awareness of the wasting of health resources |  |  |  |  |
| Your interest in planning and managing health services |  |  |  |  |

**3.6 After the experience, to what extent did you feel valued by your school of specialisation?**

Not at all; Slightly; Fairly; Very much

**3.7 Did you maintain contact with the African hospital / context where you worked?**

Yes; No

**3.8 After the elective experience, did you have any other work experience in LMICs?**

Yes; No

**3.9 Do you plan to have other work experiences in LMICs in the future?**

Not at all; Slightly; Fairly; Very much

**3.10 After the experience, have you maintained contact with DwA?**

Yes; No
